# Conditions for Successful Learning of Primary School Pupils in the Context of Integrated Education: A Case Study

**DOI:** 10.1007/s10780-023-09489-5

**Published:** 2023-04-08

**Authors:** Daiva Jakavonytė-Staškuvienė, Ingrida Mereckaitė-Kušleikė

**Affiliations:** Education Academy, Vytautas Magnus University, Kaunas, Lithuania

**Keywords:** Primary school pupils, Conditions for successful learning, Integrated education, Case study

## Abstract

Contemporary educational theory and practice increasingly emphasise the importance of integrated education in creating the conditions for success for primary school students. In Lithuania, as part of the 2020–2024 curriculum update and the development of general curricula, integrated education is highlighted as one of the priorities for achieving harmony between pupils’ academic achievements and the development of general competences, as well as the creation of a favourable emotional environment for pupils. This creates a strategic perspective on the content of education. However, there is a lack of research and insights into how such practices can be developed smoothly. For this reason, our research was carried out by targeting one of the Francophone institutes in southern France, which has successfully implemented an integrated education practice that enables learning for every pupil in the primary grades. The study analysed the context, i.e. the concept of the educational institution’s activities, which shows what should be done and how it should be done in order to achieve the quality of integrated education. In addition, the data were obtained through semi-structured interviews with 4 teachers at the Institute with experience in integrated education. Qualitative content analysis was used to inductively search for answers describing the essence of integrated education, the most important processes to reflect on before engaging with pupils, and to deductively identify how certain elements contribute to creating the conditions conducive to integrated education and pupils’ successful learning.

## Introduction

Defining “successful learning” is not an easy task, but depends on the educational strategy chosen, the values and content analysed, and the achievements of the students. The years 2020–2024 are a period of renewal and strategic, political and practical agreements on the content of education in Lithuania, as the content of education is being renewed from pre-school to grade 12. It was agreed that general education should be based on the practice of developing students’ general and subject competences. The general competences identified are: communication, cognitive, citizenship, cultural, digital, creative, social-emotional and healthy life-style competences. The aim is to reinforce the development of personal qualities and values, focusing on dignity, trust, empathy, responsibility, democratic culture, self-confidence, will and resilience. To develop all these personal qualities, the school environment should first and foremost be a safe environment that motivates pupils to learn; one that children want to be in. The aim is *to create the conditions for each pupil to achieve at higher levels, to provide a solid and sustainable foundation of knowledge* (based on the document Guidelines for updating the Framework Programmes, 2019). Successful learning can therefore be defined as competency-based learning that focuses on subject performance and personal development (Herzog-Punzenberger et al., 2017). Particularly in the context of recent years, when education is organised in the context of the COVID-19 pandemic, there is an emphasis on targeted support to the learner for successful learning (based on European Commission publications Teachers and school leaders in schools as learning organisations: report of the ET2020 Working Group Schools, 2018, Blended learning in school education, 2020), because it depends on the pupil’s emotional state (UNESCO, 2018, 2021). This support is not only understood as the teacher’s support for the learner in a particular subject, but also as community support (Acree et al., 2017; Education Development Trust. *Successful school leadership*, 2016), provision of the necessary tools (especially in blended learning contexts combining face-to-face and distance learning). In this context, the role and competence of the teacher, the ability to assess the situation, to stimulate students’ learning, to create a motivating learning environment based on innovation, the development of students’ competences, and the practice of dialogical activities are all emphasised.

## Review of Related Studies

### A Foundation or Vision for Successful Learning (Why Learn?)

The practice of integrated education creates the conditions for transforming the school as a community and the educational process in each classroom (Acree et al., 2017; Education Development Trust. *Successful school leadership*, 2016). The teacher in this kind of education helps children to answer the questions of *how to learn, who needs it and why* (Azis, 2015; Black & Wiliam, 2018; Monteiro et al., 2021). If sustainability is the goal, the whole school community is consulted on the vision of integrated education. The choice of model is discussed and consulted within the educational institution itself (according to Jakavonytė-Staškuvienė, 2017, 2021a, 2021b). The vision for integration is about how content will be integrated, not just in one subject but across the curriculum/all subjects, what content or part of content will be integrated (according to Alexander, 2020; Arrow et al., 2019; Duibhir & Cummins, 2012; Håland et al., 2021; Skaftun et al., 2021). Children find learning more engaging and interesting when it includes elements of at least two subjects (Integrated Learning in the Classroom, 2010). If it is decided to teach several languages in an integrated way in a school, consideration should be given to how the different groups of children (by language) will be organised, i.e. the organisation of the groups, the classrooms, the number and distribution of learners (Basso, 2014; Jakavonytė-Staškuvienė, 2017). In the classroom, it is important to observe and analyse how summative assessment relates to formative assessment. Assessment is understood as a process of communicating learning, and formative assessment should not be seen as a good thing and summative assessment as a bad thing, as they are interdependent (according to Houston et al., 2017). Balanced assessment in primary education therefore means integrating formative and summative assessment into the teaching process.

### Content (What is Being Learned?)

When thinking about content, it is important to anticipate which topics to integrate will be foundational and which subjects will form the basis of the integrative subject area. It is also worth thinking about the activities that will be used to build this, the research that the children themselves will be able to do, the challenges they will have to overcome and what they will have to achieve. The pedagogical strategies to be implemented in the activities, the learning resources available, the main educational approaches or assessment strategies and methods, are also part of the design of the integrated content (Basso, 2014; Shulman, 1986), recommends inquiry-based, cooperative learning. The most common way to deal with these situations is to work in pairs or small groups. Integrated tasks should teach how to navigate life situations and strengthen personal skills based on experience. Good educational practice is linked to pupils’ willingness to learn, self-confidence, analysis of mistakes and expressing their opinions (Barnes et al., 2017; Black & Wiliam, 2018; Bronkhorst et al., 2020; Pasquini, 2021). Pupils understand the learning intentions/expectations and know the criteria for the task. Learners receive effective feedback based on pre-discussed and agreed criteria. Focus on concepts and their use in different contexts in the design of what is to be learned; emphasis on projects/assignments; flexible organisation of activities, learner focus in activities (pair and small group work); use of authentic sources beyond textbooks (Integrated Learning in the Classroom, 2010). A conception of subject content that links knowledge to the epistemology of knowledge and reflective learning knowledge based on problem-solving practice (Orange, 2012), activities based on dialogue (Håland et al., 2021; Skaftun et al., 2021; Wagner et al., 2020). When learning a particular subject/experience, it makes sense to organise the activities in such a way that children learn to think like experts/inventors in a particular field. Educational activities could become a kind of children’s research practice/laboratory, based on the approach of a linguist/historian/mathematician etc. (Vinatier, 2009, 2020). Subject discussions are didactic situations conducive to the development and exploration of students’ critical thinking and the conditions for its development (Pastré, 1999; Pastré et al., 2006; Pasquini, 2021; Vergnaud, 1990). What matters is the learning that children initiate and the educational content they create. When thinking about the basis for the development of generic competences, it is appropriate to organise student conferences (Benvegnen et al., 2021; Mottier Lopez et al., 2019; Saillot, 2019), based on the analysis of students’ work, a critical approach and oral expression of ideas. It is prepared in advance and students have the opportunity to discuss and speak (Black & Wiliam, 2018; Monteiro et al., 2021; Pasquini, 2021). It is important that students learn to reflect on their experiences before, during and after the activity. This allows children to reflect on what they already know, what skills they can apply in other contexts, and to see what they can do to develop their skills further. The success of such tasks is linked to the learner’s academic and personal progress, good emotional well-being (according to Florin & Guimard, 2017).

### Creating an Environment Conducive to Learning (How Do I Learn?)

#### Physical Environment

An important issue is the school’s space and environment, which directly determines the well-being of pupils. School buildings built many years ago reflect the ideology of the time and often hinder, rather than help, the development of modern, integrated education (Dizerbo, 2016; Durpaire & Mabilon-Bonfils, 2017; Musset, 2012). Seating behind each other, with pupils seeing each other’s backs instead of their eyes, does not lead to integrated content and collaboration. Such environments encourage direct pupil guidance and frontal, control-based teaching. Today, construction practice is more dependent on the environment, aesthetic or financial possibilities (according to Hébert & Dugas, 2017). Thus, research shows that students in large schools feel more insecure in unsupervised spaces, large schoolyards, toilets, playgrounds (according to Hébert & Dugas, 2017). These spaces are important in the context of integrated education under the “school without walls” principle, where children are encouraged to make use of all the spaces available in the school, but the challenge for the teacher is how to manage a large classroom and ensure the safety of all the students. If there is a teaching assistant or a second teacher in the classroom (as most private schools in Lithuania do), it is easier to create such conditions, but it is much more difficult in a public school, as few schools have teaching assistants. This is another reason why integrated education is less common in educational practice. Researchers Barrett et al. (2015) identified factors in the physical environment of the school classroom that influence children’s learning and well-being in the classroom: factors related to students’ comfort (lighting, noise/distraction, temperature, air quality, personal space), factors that enable students to meet their cognitive needs (clearly identifiable, personalised elements, memorisation, strategies for doing activities, teacher support/assistance), and aesthetic factors (harmony of colours and placement of different classroom elements/furniture, adaptability to active activities, mobility of furniture). Again, these listed components are important when planning integrated activities, as the tools should be accessible to the students and the layout should be such that everyone feels comfortable working in pairs or small groups. In Lithuania, this is sometimes difficult to do in classrooms with a large number of pupils and a small amount of space. In addition, if the teacher pays too little attention to ventilation in a small environment, even the air quality can be poor. In 2020, 11 schools in Vilnius had their air tested (document *Air quality in Vilnius schools substandard*, 2020). In one school, the highest CO2 concentration recorded was 5152 ppm, compared to the legal limit of 1500 ppm. This is to the detriment of children’s health: they suffer from a higher incidence of respiratory diseases, asthma attacks, allergic reactions, and their educational performance: they are more likely to become irritable, drowsy, and, most importantly, to be less able to focus their attention and to carry out tasks that require them to think more.

**Fig. 1:**
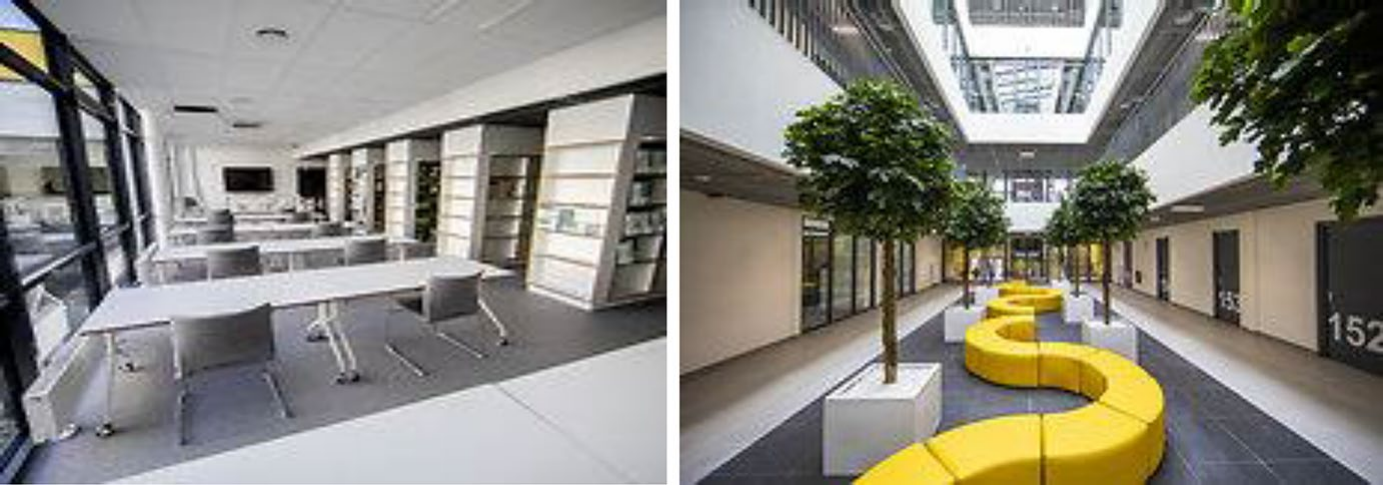
Examples of mobile school spaces. *Source* https://www.15min.lt/naujiena/aktualu/lietuva/naujoji-gabijos-progimnazija-baigiamos-paskutines-smulkmenos-bet-moderniausia-iranga-jau-yra-56-1367244#galerija/207490/5513856

The space could be modular and flexible (Dizerbo, 2016; Durpaire & Mabilon-Bonfils, 2017; Musset, 2012), so that it can be adapted to the size of the group, to new teaching methods, to extracurricular activities, and to encourage social interaction and allow students and teachers to work as a team. In addition, ICT resources and teleworking in school settings should be taken into account. In the Lithuanian context, private schools are being established in highly innovative environments, often with integrated education ideas; most private schools are located in non-standard environments, allowing them to change the size of the spaces according to their needs (upsizing or downsizing if necessary). In recent years, public schools have also been built in a modern style, with a large number of mobile spaces that can be used during the educational process. Here are the mobile spaces of the most recent public school in Vilnius in 2020 (Fig. 1).

Two new public schools built in the capital of Lithuania in 2020. In the next 5 years, 5–8 new public schools are planned. However, most of Lithuania’s public schools were built more than 30 years ago, which means that educational environments are often designed differently, without mobile spaces. Therefore, innovation in education requires a lot of effort to break down walls and increase classroom space so that integrated education activities can be developed in a cross-curricular way, with separate subject spaces. Of course, if a teacher is willing and enterprising, he or she can create such an environment on his or her own, but he or she needs to put in more effort.

#### A Safe Emotional Environment

Integrated education emphasises individualised active learning activities, where the learner is actively involved in the educational process. It is important that the teacher values the diversity of learners, supports all learners, and works with all learners (taking differences into account). This should promote learning in both academic and practical contexts and in a supportive social and emotional context for all learners (according to Chauvière, 2018; Andersson & Palm, 2018; Chetty, 2019). The educational content is updated to address issues relevant to the pupils, the children work actively in a collaborative way, discussing, solving, creating, experimenting, everyone has a say, and they feel safe because of it (Håland et al., 2021; Skaftun et al., 2021; Wagner et al., 2020). This way, the content is best absorbed and retained in memory for the longest time. This progresses both the individual’s autonomy and cognitive abilities/achievements in science.

In summary, the success of pupils’ learning in the context of integrated education could be organised according to a number of factors that we have identified in the theoretical model of integrated education, which includes academic scientific and innovative pedagogical levels and an environment conducive to learning (see Fig. 1).

**Fig. 2:**
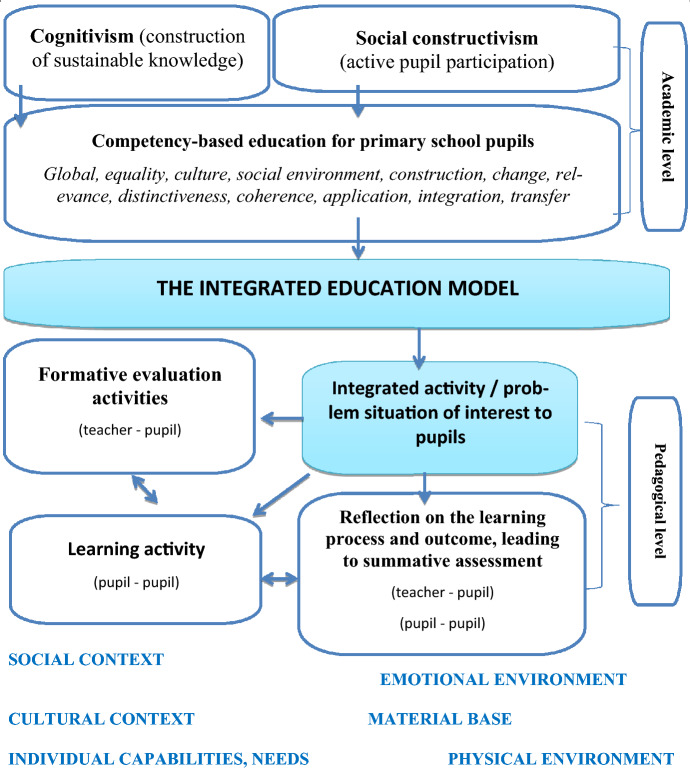
A model of integrated education for primary school pupils, combining academic scientific and innovative pedagogical levels and an environment conducive to successful learning (compiled by the authors)

From the data in Fig. 2, we can see that integrated education is distinctive in that it can encompass the general and subject-specific competences of pupils, which are prerequisites for academic success, and in that the nature of the activity involves a problem situation that is of interest to the child, which is created by taking account of the individual child’s needs; the social, cultural, emotional context, and the material and physical environment.

## Methods

In view of the theoretical background of the topic, when Ingrid Mereckaite was preparing to develop an integrated content in a Lithuanian primary school, where different competences of several subjects are covered, with an emphasis on the integrative part of the language, an exploratory qualitative case study was carried out in order to investigate the conception of the integrated education of one of the Institutes of Integral Language Teaching in the southern region of France (the “Institute of Francophony”). The study aimed to investigate the qualitative aspects of integrated education that contribute to students’ success, such as: processes, cross-curricular teacher collaboration and pupil collaboration, the creation of a supportive environment for students, methods and tools.

### The Context of the Educational Establishment Where the Study was Carried Out

The Francophonie Institute operates on the principles of an integrated approach that encourages language learning through exploration and positive learning. Each integrated activity module developed is a good practice developed in the educational establishment. Training courses are developed by training other teachers from different countries, using innovative digital solutions adapted to the learner’s needs. Mobile apps, videoconferencing, webinars help to transfer learning in a way that works for the learner. Live contact, distance or blended learning is one of the options available for integrated learning. Throughout the year, the Institute Francophonie organises training courses for French teachers and other learners from all over the world. Characterised by pedagogical excellence, innovation and interculturalism, these training courses allow teachers to share their experiences and deepen their pedagogical practice while exploring and getting to know a unique environment. A highlight of the Institute’s training programmes is the summer courses in July and August. Each year, around 1500 students from 91 countries study at the Francophonie Institute. The Francophonie Institute invites teachers selected by the Research Council to work for it on a 1- or 2-year contract. Teachers must be highly motivated, have a high level of subject competence and hold a certificate in integrated education. Each teacher may work in one or two modules of integrated content. Each year the programme and integration (horizontal, vertical) of the modules is reviewed and reconsidered. Each integrated module consists of five key principles, which focus on: educational philosophy, content, methods, environment, and student well-being. Examples of the concept of two modules are given below.

The Institute’s practices, as outlined in Table 1, are based on a framework of personal development and learning incentives, underpinned by the development of emotional competences. Such practices would help to achieve personal progress for every learner in any new subject. It is important that each learner’s autonomy is fostered, taking into account his or her existing competences and social context. This means that the tools and means of support will be different, according to individual needs and experience. This practice, based on positive dialogical activities, is also recommended by other researchers (Håland et al., 2021; Skaftun et al., 2021; Wagner et al., 2020).

**Table 1:** Example of the concept of the *Emotion and Motivation Education as a Learning Lever module* at the Francophonie Institute for Integrated Education

Emotions and motivation as levers for learning This course aims to develop students’ motivation, self-confidence and commitment to language learning. It is a participatory learning course that alternates practical experiments with theoretical material, and its approach is based on the latest research in positive psychology, the science that studies the optimal functioning of individuals, groups and institutions (Gable & Haidt, 2005). Students learn through playful experimentation, using scientifically validated methods to develop psychosocial skills, such as emotion management or positive relationships, while achieving quality outcomes. The essential aspects of integrated education are applied in the module. 1. Emotion management: understanding and learning to manage the expression of emotions It is important to understand the impact of emotional education on readiness to learn and to teach students to recognise their emotions through a range of exercises that are integrated into a range of activities aimed at understanding the role of emotions and the ability to recognise, express and manage them 2. Expressing and developing pleasant emotions in language learning The educational process uses scientifically validated methods and tools to regulate unpleasant emotions and promote students’ pleasant emotions. If a child is afraid to read aloud or afraid to talk when the surroundings are quiet, he/she learns to overcome his/her fears slowly by talking about them through indirect activities. For example, reflection, emotion cards, pre- or post-activity talk, keeping a personal diary 3. Fostering motivation in the classroom When teaching students in any classroom, identify the factors that motivate children to learn the language. The aim is to understand the components of motivation according to Deci and Ryan’s (2008) theory of self-determination and to understand the three main psychological needs that underlie motivation: *autonomy, competence and social context*. Taking into account all three of these needs, a system of tasks and activities is designed for a specific group of learners 4. Developing motivation and goal achievement Together with the children, they talk about the motivation system used in the classroom and the aims of education. It is important that both the educational objectives and the motivational methods are set by each individual pupil and that they discuss with the teacher the steps they will take to improve. These elements are not only applied in the educational process, but are also systematically discussed with the pupil in order to analyse how he/she is doing, how much effort has been made, etc 5. Discussion of activities The educational process includes feedback on the methods used during the week, useful tools, asking for children’s opinions, and the creation of self-motivation and positivity memos	

The module in Table 2 highlights the Institute’s contemporary didactics, which focuses on the active engagement of the learner.

**Table 2:** Example of the concept of a module on *Project-Based Pedagogy with Activity-Based Learning* at the Institute of Francophonie for Integrated Education

Project-based pedagogy with a focus on active engagement This module highlights some aspects of task-based pedagogy and learning by doing. The aim is for students to engage in purposeful learning situations, i.e. their actions are directed towards solving a problem and/or creating an ‘object or product’. This requires the use of a number of resources: internal (culture, knowledge, experience, etc.), external (methodological tools, protocols, worksheets, documents, other resources, etc.), which the teacher should prepare for the pupils The aim is to develop practices that are relevant to the realities of modern life, and to focus learning on the goals to be achieved, without losing sight of the process. At the end of the module, students present the work they have produced during the practice and discuss the process. The essential aspects of integrated education are applied in the module: 1. Linking different approaches to teaching a particular subject or several subjects Didactic activities offered to pupils, where goals, objectives, expected results and the methodology for achieving them are defined in consultation. A particular active learning method or strategy chosen by the pupils is adopted 2. Motivating and engaging activities Students learn to apply theory in practical activities through active learning, analysis, research and drawing conclusions. In addition, activities are chosen to motivate pupils to learn, as motivation is recognised as a driving force for learning 3. Dividing subject content into parts and linking the parts together Students are introduced to subject content by showing how this content is presented in other subjects, how it is linked between subjects, what is different and why. In addition, pupils are introduced to collaborative roles, where each person learns not only to fulfil his or her assigned role, but also to assimilate the content for which the other members of his or her team are responsible 4. Managing class heterogeneity Project-based pedagogy and *problem-solving techniques* are often used. The teacher, when selecting an activity for a particular child, reflects on the student’s personal experiences in order to personalise and contextualise the task. Teachers look for ways to enable pupils to work independently and collaboratively 5. Presentation of the project or other complex tasks completed The basis for learning is the independent learner. Students are encouraged to develop projects using a task-based methodology, including: objectives, planning, criteria for quality work (as a result), format, timeframe, progression, complexity and systematicity of tasks, summarising and presenting the result. It is important to record and reflect not only on the results, but also on the process, the discussions that arise, the problematic situations and the solutions	

The content in Table 2 shows which didactic tools contribute to a student’s success in integrated education, which is a self-directed, goal-oriented, multi-tasked, personal learning path for each child. It is important that the subject content is discussed, its interdisciplinary nature, the knowledge base where it can be applied in practice, and that the main activities involve a series of projects that are developed from plan to result.

### Study Method

The study was carried out in June 2019, during the “Linguistics Week” organised by the Institut de la Francophonie de France du Sud. As the activities of the learning week were carried out on the basis of an integrated education, using the environment to stimulate the motivation of the learners, creating a favourable learning environment.

The semi-structured interviews with the study participants addressed 5 qualitative themes related to successful learning in integrated education: processes to consider when planning integrated activities (research question 1); collaboration between teachers from different subjects (research question 2), collaboration between students (research question 3), creating a supportive environment for students and the skills they develop (research question 4), and methods and tools used in an integrated education context (research question 5) (Lafontaine, 2016). The rationale for each of the semi-structured interview questions, i.e. what was sought in formulating them, can be seen in Table 3.

**Table 3:** Semi-structured interview questions to explain integrated education as a sub-framework for successful learning

Question	Purpose
What is the most important thing in integrated education?	Find out what agreements and processes are in place to organise integrated education
How do you communicate and collaborate with colleagues during periods of planning and implementation of integrated education?	Identify how the prevailing communication culture affects teachers’ interpersonal communication
How do you create opportunities for pupils to work together?	To clarify the organisation of the work of educational activities in terms of pupils’ cooperative expression
How are students’ skills developed in a learning environment? What do you observe in the pupil’s abilities in integrated activities?	To find out teachers’ attitudes towards creating a learning environment; and to identify the ways in which learning environments are created Identify what teachers focus on most, and what pupils’ abilities are identified
Which methods or tools motivate pupils the most?	Find out teachers’ views on the most motivating tools or techniques for pupils

The 4 participants in the study were teachers working in the Institute of Francophone Education in the South of France, who are highly competent in the field of content development in integrated education. The participants were informed in advance of the themes, which were discussed in the same order in all four interviews. Each interview lasted approximately 1 h and all interviews were tape-recorded. Each participant had the same interview conditions to ensure objectivity and complete anonymity during all interviews. The names of the teachers interviewed have been changed to Julien, Jeanne, Claire, Yann. The data of the participants in the study by type of activity and competence are presented in Table 4.

**Table 4:** Background information on the study participants

Participant pseudonym	Gender	Years of work experience (years)	Scientific or pedagogical title	Functions in the Institute	Interview duration (min)
Julien	Male	10	Doctor of Education, teacher	Monitoring Team Leader, Teacher	51
Jeanne	Female	9	Master’s degree in Linguistics, teacher	Training organiser, programme administrator	43
Claire	Female	18	Expert teacher	Teacher	45
Yann	Male	25	Doctor of Linguistics, Educator	Programme manager, member of the Scientific Council, teacher	57

### Data Analysis Method

Qualitative content analysis (Elo & Kyngäs, 2008; Tirri et al., 2021) has been used to inductively search for answers describing the essence of integrated education, the most important processes to reflect on prior to the activity with the pupils (research question 1), and to deductively identify how certain elements contribute to the creation of a favourable environment for the integrated education and the pupils’ successful learning (research questions 2–5). The unit of analysis was a unit of meaning consisting of one or more sentences answering one of the research questions. In the inductive analysis, the units of meaning were compared and abstracted into two main categories (monitoring and educational process) (see Table 5). For the deductive analysis, the following units of meaning were extracted: agreements, responsibilities, place, time, form, learning from each other, encouraging verbal expression, developing personal qualities, ICT tools, personalised activities, group work, project activities (see the analytical fields of questions 2 to 5). The researchers coded the teachers’ reflections according to the categories and analysed the extent to which they reflected their experience of integrated education. According to Žydžiūnaitė and Sabaliauskas (2017), the aim of a case study is to describe phenomena by systematizing and summarizing the research material, to reveal the meanings people give to these phenomena. In the case of our study, the question was what and how should be done in order to bring the key provisions of integrated education to an educational institution that did not apply or rarely applied integrated education. A convenience case sample (Baškarada, 2014; Thomas, 2021; Yin, 2018), was used, with research participants selected from an institution that had qualitatively developed practices of integrated education.

**Table 5:** Conditions for integrated education to create a successful learning environment

Study question	Category	Example of an extract from an interview
What is the most important thing in integrated education?	Educational process (N = 4)	“Integrated education is the foundation of holistic education, the most important principle of a modern school” (Yann) “We aim to improve the quality of teaching and learning through evaluation. The focus is on the benefits of integrated education, <…> learning competences, in order to build students’ self-confidence and create opportunities for personal development” (Julien) “The quality of integrated education depends on communication and cooperation between teachers” (Claire)
	Monitoring (N = 7)	“Every year, a performance evaluation plan is drawn up. <…> We carry out a broad assessment, which leads to the selection of indicators for the in-depth assessment, the design of the research methods, the analysis of the results, and the drawing of conclusions and recommendations” (Julien)
How do you communicate and collaborate with colleagues during periods of planning and implementation of integrated education?	Agreements (N = 4)	“The most important thing is to talk about values, about what our goal is. I think the most important agreement is a goal that motivates all teachers” (Claire) “The most important thing is to stick to your role and the agreed action plan” (Yann)
	Responsibilities (N = 4)	“This can be one of the hardest parts of the phase, because at the beginning all the teachers have a lot of enthusiasm, and then the workload and the routine, it reduces that. You have to be open and objective” (Julien) “A teacher has to know himself well, know his strengths. This is the responsibility he should take upon himself” (Jeanne)
	Location (N = 4)	“School cafeteria, sometimes a workroom (a workroom is a teacher’s room with individual and group work space)” (Julien), (Jeanne)
	Time (duration) (N = 3)	“It’s hard to say, but at least 50 min every week” (Claire)
	Form (N = 3)	“Mostly informal, depending on interpersonal relationships” (Julien) “You could say dynamic, for me the most important thing is to have a good feeling” (Claire)
How do you create opportunities for pupils to work together? How do you develop pupils’ skills in a learning environment? What do you observe in the pupil’s/students’ abilities during integrated activities?	Learning from each other (N = 3)	“Proactively offering ideas and suggestions” (Julien) “Rallying other students around you” (Claire) “Willingness to help, to take the initiative” (Jeanne)
	Encouraging oral expression of thought (N = 3)	“We are always trying to give the child the opportunity to express themselves. This includes speaking out, listening to opinions. It’s an opportunity for the child to implement and propose ideas for change” (Claire)
	Developing personal qualities (N = 4)	“Perseverance, courage, action, good learning” (Julien) “Originality, quickness, activity, desire to improve” (Claire)
Which methods or tools motivate pupils the most?	ICT tools (N = 5)	“Computers and tablets in themselves motivate children to act, no matter how difficult the task” (Jeanne)
	Personalised activities (N = 2)	“Thinking maps are very helpful, they empower children to act authentically” (Claire)
	Group work (N = 2)	“The group work method brings out a lot of different qualities in the students, both positive and negative, because it is demanding and there are awkward situations” (Julien)
	Project activities (N = 2)	“The more adventurous students raise discussion questions and look for original ideas” (Yann)

## Results

By analysing interview data from French teachers with integrated education practices, we have developed detailed descriptions of their practices to help you understand how teachers create integrated education environments that support students’ learning success. A summary analysis of the empirical research data according to the identified research questions and categories and the sample statements that illustrate them is presented in Table 5.

### Priorities for Integrated Action Planning

When discussing the essential elements of integrated education, the study participants highlighted aspects such as the *planning and monitoring of the educational process*.

#### Educational Planning

It is important that, when designing integrated activities, the content is planned by teachers working in the same classroom in a collaborative and cooperative way: “we discuss the possibilities of integrated education” (Julien), and choose ways of working that “improve the well-being of the pupils during the educational process” (Jeanne). These practices are in line with the definition of good educational practice, which reflects on the well-being of the pupils, the provision of targeted support for the learner (according to European Commission publications *Teachers and school leaders in schools as learning organisations: report of the ET2020 Working Group Schools*, 2018; *Blended learning in school education*, 2020; UNESCO, 2018, 2021).

### Monitoring

Following a detailed analysis of the monitoring reports, further educational planning is carried out “in line with the recommendations, through group action plans” (Claire). In order to organise quality integrated education, the school should have a clear internal monitoring system, acceptable to the teaching community, based on teamwork, i.e. jointly developed action plans. It is also important that each teacher’s competences are first and foremost his or her own, and that the teacher’s responsibilities in relation to integrated activities depend on them: “an evaluation is carried out at the beginning of each school year and this information is used as a guide” (Julien). Teachers usually commit themselves to the area in which they are most advanced, and, in cases where a teacher’s self-assessment indicates a lack of competences, to learning and development in that area. The ideas expressed by the participants in the study on monitoring are very close to the researchers’ view that the main agreements on integrated education are made within the educational establishment (Acree et al., 2017; Education Development Trust. *Successful school leadership*, 2016; Jakavonytė-Staškuvienė, 2017, 2021a, 2021b). Importantly, a culture of “living, changing and renewing” planning is essential when working in an integrated education context: “the plan is discussed once a month, to write down changes and other proposals” (Claire). One of the challenges is the personal responsibility of each teacher to commit to and honestly reflect on his/her own process and results, as this will determine the quality of the monitoring of educational content.

### An Environment Conducive to Integrated Education and Pupil Success

The learning environment is facilitated by *cooperative approaches between teachers* and *a focus on the success of each pupil*.

*Ways of promoting cooperation between teachers* include agreements, division of responsibilities, place and time of communication and cooperation, and format.

### Agreements

A participant in the study stressed that agreements vary, “depending on the group you work in, because people are very different, you could say they are different every year” (Julien). This means that the needs and possibilities of each group of learners are taken into account. Another participant stressed the importance of getting to know each other: “if the team is known, there is no need for such a thing as an “agreement”, because you know. When you don’t know the team, then you have to be clear, because there are people who work in a destructive way” (Jeanne). Constructiveness is also important, as otherwise the result will not be achieved, especially if several teachers in the same classroom are planning to use the same teaching methods. Once a vision/direction for the implementation of integrated education has been agreed at school level, then each teacher should commit to and set specific goals and targets for what is to be achieved in integrated education. It is important that each teacher sets objectives that are appropriate to the classroom situation (Basso, 2014). It is also important that the whole community adheres to the agreements, if some teachers do not; the quality of integrated education suffers.

### Responsibilities

Important responsibilities of each teacher planning educational activities together: “It may sound ridiculous, but you need to use the same strategies as in the classroom, just to identify roles, the responsibilities of each role and then to see who can take on what personally” (Jeanne). Another participant in the study spoke similarly about the division of responsibilities: “the easiest thing to do is to have everybody say who would like to take what, and then you just divide it up” (Yann). By taking on responsibilities, teachers commit themselves to which subject content topics and methods they will be able to integrate (according to Alexander, 2020; Arrow et al., 2019; Duibhir & Cummins, 2012; Håland et al., 2021; Skaftun et al., 2021). Sharing responsibilities and committing to them is also very important in creating a culture of trust in teachers and their competence. Otherwise, if some teachers in the community fail to do so, both the quality of the integrated education process and the students’ results will suffer.

### Place and Time

In terms of location, the teachers in the study expressed that this aspect is very flexible: it could be the teachers’ workroom, it could be the corridor during break time; “This is even during the common activities, when we observe how the pupils are doing, then we immediately talk about it and improve the activity or the plan” (Yann). These statements show that the community is friendly and seeks and finds the right solution in every case, and is flexible in the way it deals with problems. This reflects a vibrant, constantly renewing organisational culture. When thinking about time, those who took part in the study said that it varies according to how much is needed in a particular case. For example, “it’s individual because different classes need different access, there are difficult groups that need more time” (Jeanne). This aspect is very important, because in order to achieve quality, this is how the process should be organised, i.e. to give time until the issue is resolved.

### Form

Forms of communication were generally reported by all participants to be more often informal and formal, “mostly free form” (Jeanne). This aspect shows that the team is well focused, knows each other, understands the common goals of the organisation, and is committed to quality education for each child, through informal, free-flowing conversations and shows that the process is based on a dialogical culture of trust and professionalism (Håland et al., 2021; Skaftun et al., 2021; Wagner et al., 2020).

*The focus on students’ success in learning was* discussed by highlighting the importance of students learning from each other, encouraging verbal expression and developing personal qualities. These important elements can be achieved through the use of a range of methods and tools, i.e. ICT tools, group work, personalised and project-based activities.

### Learning from Each Other

When it comes to when children can learn from each other, research participant Yann said that “project presentations are a great opportunity”. Indeed, pupils can help, advise and discuss activities when they work together. This is supported by research carried out by other researchers (Barnes et al., 2017; Black & Wiliam, 2018; Bronkhorst et al., 2020; Pasquini, 2021). When forming groups of pupils, it is important for teachers to think about how pupils will be grouped and what roles they might take on, because the most advanced groups are those that are made up of pupils of different abilities who are able to take on different responsibilities and commitments.

### Encouraging Verbal Expression of Thought

When talking about the aspect of integrated education where students are encouraged to express their thoughts, to argue, to express their point of view, the teachers who took part in the study stressed the importance of not only formal education but also informal education: “such opportunities are more likely to be realised in informal or extracurricular activities, as many students work at an uneven pace in the classroom” (Jeanne). This is another important aspect of the whole educational process. Consequently, when applying the elements of integrated education, it is important to talk to all educators (in formal and informal activities) to ensure that educational approaches are developed in contexts that are appropriate for children at all times. Dialogue-based practice is also identified by other researchers (Håland et al., 2021; Skaftun et al., 2021; Wagner et al., 2020) as an activity that focuses on students’ highest achievement (especially reasoned narrative, reasoning).

### Developing Personal Qualities

We would like to underline that, although different descriptions of the personal qualities of students were given, such as “dynamic, cooperative, willing to help others” (Claire), “quick to react, critical thinking” (Jeanne), all the participants in the study emphasised the student’s role as a creator, able to be active, to react and to make decisions. Hence, the practice of integrated education allows for the development of personal qualities based on the ability to produce (Pastré, 1999; Pastré et al., 2006; Pasquini, 2021; Orange, 2012; Vinatier, 2009, 2020; Vergnaud, 1990).

*ICT tools* are used in the educational process for various purposes, such as information search (“children are motivated by the quick search for information, in different electronic sources” (Claire, Yann) or reflection on the educational process (“social media, virtual communication apps: Snapchat, messenger. Sometimes we use these apps to reflect or evaluate our activities” (Julien). This means creating a modern learning environment that is relevant to the realities of life, where students use modern ICT tools that support both academic and personal learning goals (Dizerbo, 2016; Durpaire & Mabilon-Bonfils, 2017; Musset, 2012). Importantly, the use of tools in the educational process is purposeful and deliberate.

Group work is highlighted as one of the most important methods in an integrated setting, because “group work makes children more cooperative, active, has the opportunity to learn from each other, and makes them appear more relaxed, but with a higher quality of functioning” (Julien). We would like to highlight the fact that this approach is used by different teachers in different subjects. This is a very important part of the practice, as communication and cooperation skills can be developed in different contexts and tasks (Black & Wiliam, 2018; Monteiro et al., 2021; Pasquini, 2021).

### Personalised Activities

Group work allows for reflection and personalised activities, especially when group members have different tasks, each taking responsibility and committing to a result (Basso, 2014; Jakavonytė-Staškuvienė, 2017). “The most important thing is that the curriculum is relevant and the topics are relevant, so that the pupils are interested. Personalised tasks are the most important factor” (Jeanne). We would point out that it is important to take into account the personal qualities and experiences of the students. All this helps to create unique and authentic learning environments, contexts that are important for good learning and well-being in the classroom.

*Project-based activities* are also linked to personalised activities, as they allow pupils to express their experiences and areas of interest through projects, especially when they have the opportunity to propose and choose from a range of project themes. The projects can be original, offering out-of-the-box solutions to problems, etc. In addition, project activities are also linked to the oral expression of ideas, as “students demonstrate their knowledge of public speaking” (Claire) when presenting the results of a project.

## Discussion

After analysing the results of the empirical study, we can say that the conditions for successful learning for primary school students in the context of integrated education depend on two aspects: an emotionally safe and motivating learning environment and active, communicative and cooperative ways of working in the educational process, which is often the case for group work and project-based activities. To achieve all this, the educational establishment should have a community of teachers who are motivated, eager to have fun, and committed to each student’s learning (according to Acree et al., 2017; Andersson & Palm, 2018; Chauvière, 2018; Chetty, 2019; Education Development Trust. *Successful school leadership*, 2016). Research has shown that the role of the teacher is one of the most important factors and variables in the development of an integrated curriculum, as the teacher influences students (according to Hattie, 2009; Jakavonytė-Staškuvienė, 2021a, 2021b). Researchers Hattie (2009), Bressoux (2012) and Talbot (2007) stress that cognitive and motivational stimulation, participation, engagement and deliberate timing of the teacher’s task remain important in the design of classroom activities. Classroom interaction, when students work in pairs/groups/individually and when they present activities to the whole class, is an important part of the learning process. However, how smoothly this happens depends in many cases on the teacher. The teacher’s choice of task, the proposed way of working, the allocation of group members and the provision of information are all important activities for the teacher, especially when it comes to pupils in primary school. Successful education is supported by differentiated activities (also known as personalised tasks), where every student feels important and relevant and learning is challenging. This is the kind of learning that Integrated Learning is particularly conducive to (according to Integrated Learning in the Classroom, 2010; Meirieu, 2006). In primary education, given the specificities of the age range of the pupils, integrated education is organised on the basis of active activities, and in order for the activities to be successful, it is important to think about and provide children with the appropriate/necessary support, i.e. a system of methods/explanations that help them to act and to experience success (Black & Wiliam, 2018; Florin & Guimard, 2017; Monteiro et al., 2021; Pasquini, 2021). The teacher provides support by commenting on the student’s actions and suggesting how the work can be improved.

If the school is carrying out a project in an area within the framework of integrated education, there is also an agreement with the pupils and the conditions for planning, carrying out, analysing, drawing conclusions and presenting data. Usually, a project activity plan is developed and shared with all pupils in the class (according to Gégout, 2013; Connac, 2016). It is important that the project activities are properly summarised in the form of student conferences or learning celebrations (Benvegnen et al., 2021; Mottier Lopez et al., 2019; Saillot, 2019).

## Study Limitations

The main limitation of this study is the sample size. The participants in our study were teachers and content administrators from one institution that has a high-quality practice of integrated education for primary school pupils. For this reason, the data cannot be applied to the whole population, but it does show how the quality of integrated education can be achieved. We believe that the findings of the study are of relevance to educational policy makers who make decisions about the content of education, to researchers, and to school leaders or teachers who want to start applying the principles of integrated education but do not know how to do so qualitatively.

## Conclusions

Monitoring the educational process is one of the key factors underpinning the quality of integrated education. It also emphasises the use of qualitative (reflection, individual interviews) rather than quantitative monitoring parameters. Teachers, who communicate, discuss and collaborate about pupils’ learning help to create an environment conducive to learning.

Participants in the empirical study reported that collaboration between teachers in the organisation of integrated education is mostly on their own initiative and in the form they choose. It is also important to mention that all interviewees describe the development of an integrated education process as a flexible phenomenon, dependent on a group of people. Teachers tend to allocate responsibilities according to their strengths.

It is important to note that the nature of the activities in the context of integrated education focuses on active activities in which the student strives not only for academic achievement but also for the development of personal qualities. All the informants said that pupils’ cooperation is manifested in the form of making suggestions, helping each other and taking initiative. The qualities that emerged most clearly were courage, perseverance, critical thinking, dynamism, and a willingness to help others.

A supportive learning environment and a balance of tools and approaches remain essential to the success of each child’s learning. Targeted tools may include certain ICT solutions, social media, which are an integral part of children’s daily lives, both for communication and learning. The quality of education also depends on the tasks that are chosen for the children, taking into account their personal abilities and their experience and area of interest. This can include original and individual project work.
